# Physical activity and total serum bilirubin levels among insulin sensitive and insulin resistant U.S. adults

**DOI:** 10.1186/2251-6581-13-47

**Published:** 2014-04-07

**Authors:** Paul D Loprinzi, Kalen Abbott

**Affiliations:** 1 Department of Exercise Science, Donna & Allan Lansing School of Nursing & Health Sciences, Bellarmine University, Louisville, KY 40205, USA; 2 Maricopa Integrated Health System, Phoenix, AZ, USA

**Keywords:** Accelerometry, Epidemiology, Health

## Abstract

**Background:**

Total serum bilirubin has been identified as a novel biomarker for metabolic disease, with higher levels providing protection against metabolic disease. To our knowledge, only 3 studies, to date, have examined the association between physical activity and total serum bilirubin, with these studies reporting mixed findings. One potential reason for the mixed findings may be the exclusive use of self-report physical activity methodology. The purpose of this study was to examine the association between accelerometer-assessed physical activity and total serum bilirubin among a national sample of U.S. insulin sensitive and insulin resistant adults.

**Methods:**

Data from the 2003-2006 National Health and Nutrition Examination Survey were used. Physical activity was objectively-measured using an accelerometer over a 7 day period. Bilirubin levels were assessed from a blood sample. Data was analyzed in 2013.

**Results:**

After adjusting for age, gender, race-ethnicity, BMI, comorbid illness, cotinine, and poverty level, moderate-to-vigorous physical activity (MVPA) was associated with bilirubin for insulin resistant individuals (β = 0.08; p = 0.04), but not insulin sensitive individuals (β = 0.02; p = 0.38).

**Conclusions:**

MVPA is associated with total serum bilirubin levels among U.S. adults with insulin resistance. Future experimental and prospective studies are needed, with further attention focused on the mechanisms that may help to explain the association between physical activity and bilirubin.

## Introduction

It is well established that various physiological parameters, such as cholesterol, blood pressure, and glycemic control, play an important role in physical and mental health. In addition to these established risk factors, serum bilirubin is considered to be a new biomarker for various chronic diseases [1]. Research over the last two decades has shown that low levels of serum bilirubin are associated with increased risk of cardiovascular disease [2], metabolic syndrome [3], type 2 diabetes [4], stroke severity [5], certain cancers [6], autoimmune disease [7], and psychiatric disorders [8]. Bilirubin may modulate risk of these diseases by, for example, reducing lipid peroxidation and mitigating inflammation [9,10].

Given the antioxidant properties of bilirubin [10], cost-effective strategies to increase bilirubin levels are needed. One potential modifiable behavior to increase bilirubin levels is physical activity; regular participation in physical activity has been shown to increase antioxidant enzyme and coenzyme activities [11]. However, at the time of this writing, we are aware of only three studies examining the association between physical activity and bilirubin levels [12-14], with these studies demonstrating mixed findings.

To increase our knowledge base of the potential association between physical activity and serum bilirubin levels, the purpose of this study was to use data from the 2003-2006 National Health and Nutrition Examination Survey to examine the association between physical activity and serum bilirubin levels among U.S. adults. To overcome the self-report physical activity limitations of previous studies on this topic, this study will employ an objective-measure of physical activity (i.e., accelerometry). Given the protective effect of high total serum bilirubin levels in preventing diabetes [15], focus of the present study will be to examine the association between accelerometer-assessed physical activity and total serum bilirubin levels among insulin sensitive and insulin resistant adults.

## Methods

### Study design and participants

Data from the 2003-2006 National Health and Nutrition Examination Survey (NHANES) were used. NHANES is an ongoing survey conducted by the National Center for Health Statistics. NHANES evaluates a representative sample of non-institutionalized U.S. civilians, selected by a complex, multistage probability design. Briefly, participants are interviewed in their home and then subsequently examined in a mobile examination center. NHANES data is publically available data, with the authors using NHANES data for secondary analyses. All procedures for data collection were approved by the National Center for Health Statistics ethics review board, and all participants provided written informed consent prior to data collection. For the present analyses, 2,070 participants provided data for the study variables.

### Determination of insulin sensitivity and insulin resistance

The Homeostasis Model Assessment (HOMA) was used to evaluate insulin resistance using the following formula: fasting serum insulin (uU/mL) × fasting plasma glucose (mmol/L) / 22.5 [16].

Participants were classified as insulin sensitive if their HOMA score was ≤ 2.6, with a HOMA score > 2.6 used to denote insulin resistance [17]. The Tosoh AIA-PACK IRI, a two-site immunoenzymometric assay, was used to measure blood insulin levels, with glucose measured spectrophotometrically. Details of the assessment of insulin and glucose have been previously described [18].

### Determination of total serum bilirubin

Total serum bilirubin was measured in mg/dL using the LX20, which uses a timed-endpoint Diazo method to measure the total concentration of bilirubin. Details have been previously described [19].

### Determination of physical activity

2003-2006 NHANES participants were asked to wear an ActiGraph 7164 accelerometer during all activities, except water-based activities and while sleeping. Prior to the participant’s examination, accelerometers were initialized to collect data in one minute time periods. Estimates for time spent in moderate-to-vigorous physical activity (MVPA) were summarized in 10-minute bout periods [20]. Activity counts per minute of ≥ 2020 were used to denote MVPA intensity [21]. Nonwear was defined by a period of a minimum of 60 consecutive minutes of zero activity counts, with the allowance of 1-2 minutes of activity counts between 0 and 100 [21]. For the analyses described here, only those participants with at least 4 days with 10 or more hours per day of monitoring data were included in the analyses [21].

### Covariates

Information about age, gender, and race-ethnicity were obtained from a questionnaire. As a measure of socioeconomic status, poverty-to-income ratio (PIR) was assessed, with a PIR value below 1 considered below the poverty threshold. The PIR is calculated by dividing the family income by the poverty guidelines, which is specific to the family size, year assessed, and state of residence. Serum cotinine was measured as a marker of active smoking status or environmental exposure to tobacco (i.e., passive smoking). Serum cotinine was measured by an isotope dilution-high performance liquid chromatography/atmospheric pressure chemical ionization tandem mass spectrometry. BMI was calculated from measured weight and height (weight in kilograms divided by the square of height in meters). A comorbidity index count variable was created [22]. Participants were classified as having 0 or 1+ comorbidities based on self-report of the following chronic diseases/events: arthritis, coronary heart disease, heart attack, congestive heart failure, stroke, cancer, emphysema, chronic bronchitis, asthma, or hypertension.

### Data analysis

All statistical analyses (STATA, version 12.0, College Station, TX) accounted for the complex survey design used in NHANES by using survey sample weights, clustering, and primary sampling units. Data was analyzed in 2013. Means and standard errors were calculated for continuous variables and proportions were calculated for categorical variables. An adjusted Wald test was used to examine differences for continuous variables and a design-based likelihood ratio test was used for categorical variables. Consistent with other studies [13], bilirubin levels across tertiles of MVPA were examined. The median bilirubin of the MVPA tertiles was fit as a continuous variable to estimate the trend across MVPA tertiles in a linear regression model. To further examine the association between MVPA and bilirubin (outcome variable), multivariable linear regression analysis was employed. Two models were computed: one for insulin sensitive participants and another for those with evidence of insulin resistance. Models controlled for age, gender, race-ethnicity, BMI, comorbidity index, cotinine, and poverty level. A *p* < 0.05 denoted statistical significance for all analyses.

## Results

Table 1 reports study variable characteristics among insulin sensitive and insulin resistant participants. Insulin resistant, compared to insulin sensitive participants, were older, more likely to be male, less likely to be of non-Hispanic white origin, had a higher BMI, were more likely to be in a worse poverty level, were more likely to have comorbid illness, engaged in less MVPA, and had lower (worse) bilirubin levels.

**Table 1:** Characteristics of insulin sensitive and insulin resistant participants, NHANES 2003-2006

	**Mean/Proportion (95% CI)**		
**Variable**	**Insulin sensitive**	**Insulin resistant**	**P-Value**^**†**^
	**(HOMA ≤ 2.6) (n = 1,238)**	**(HOMA > 2.6) (n = 832)**	
Age, yr	47.3 (46.1-48.5)	49.6 (48.1-51.1)	0.002
% Male	45.0 (41.4-48.5)	53.7 (50.3-57.2)	0.001
Race-Ethnicity, %			0.0007
Mexican American	7.2 (4.8-9.6)	10.1 (6.7-13.5)	
Non-Hispanic White	75.0 (69.8-80.1)	70.9 (65.3-76.6)	
Non-Hispanic Black	8.4 (5.9-11.0)	12.1 (8.7-15.5)	
Other	9.2 (6.6-11.9)	6.6 (4.4-8.9)	
Body Mass Index, kg/m^2^	26.6 (26.3-26.9)	33.1 (32.4-33.9)	<0.0001
Cotinine, ng/mL	55.5 (46.4-64.6)	49.8 (40.0-59.6)	0.39
Poverty-to-Income Ratio	3.3 (3.1-3.4)	3.1 (2.9-3.2)	0.01
Comorbidity Index, %			<0.0001
0 Comorbidities	53.1 (49.6-56.7)	34.8 (31.5-38.1)	
1+ Comorbidities	46.8 (43.2-50.3)	65.1 (61.8-68.4)	
MVPA, min/day	7.4 (6.6-8.2)	5.0 (4.0-6.0)	0.0001
Bilirubin, mg/dL	0.79 (0.76-0.81)	0.76 (0.73-0.78)	0.01

^†^An adjusted Wald test was used to examine differences for continuous variables and a design-based likelihood ratio test was used for categorical variables.

Table 2 reports bilirubin levels across tertiles of MVPA for both insulin sensitive and insulin resistant individuals. For both insulin sensitive and insulin resistant individuals, there was a dose-response relationship between MVPA and bilirubin, with individuals engaging in more MVPA having higher bilirubin levels.

**Table 2:** Mean (95% CI) bilirubin levels across physical activity tertiles among insulin sensitive and insulin resistant participants, NHANES 2003-2006

	**MVPA Tertile 1**	**MVPA Tertile 2**	**MVPA Tertile 3**	**P-Value**^**†**^
	**(0 min/day)**	**(2.2 min/day)**	**(16.6 min/day)**	
	**Insulin Sensitive Participants (HOMA ≤ 2.6) (n = 1,238)**			
Total Bilirubin, mg/dL	0.74 (0.71-0.78)	0.82 (0.77-0.87)	0.83 (0.78-0.86)	0.001
	**Insulin Resistant Participants (HOMA > 2.6) (n = 832)**			
Total Bilirubin, mg/dL	0.73 (0.70-0.76)	0.77 (0.71-0.82)	0.81 (0.75-0.86)	0.03

^†^The median bilirubin of the MVPA tertiles was fit as a continuous variable to estimate the trend across MVPA tertiles in a linear regression model.

MVPA = Moderate-to-vigorous physical activity.

Table 3 shows the multivariable linear regression analysis further delineating the relationship between MVPA and bilirubin (outcome variable). After adjusting for age, gender, race-ethnicity, BMI, comorbid illness, cotinine, and poverty level, MVPA was associated with bilirubin for insulin resistant individuals (β = 0.08; p = 0.04), but not insulin sensitive individuals (β = 0.02; p = 0.38).

**Table 3:** Multivariable linear regression analysis examining the association between physical activity and bilirubin (outcome variable) among insulin sensitive and insulin resistant participants, NHANES 2003-2006

	**Δ (95% CI) in Bilirubin (mg/dL)**^**†**^			
**Variable**	**Insulin sensitive**	***P***	**Insulin resistant**	***P***
	**(HOMA ≤ 2.6) (n = 1,238)**		**(HOMA > 2.6) (n = 832)**	
MVPA^**‡**^	0.02 (-0.02 to 0.07)	0.38	0.08 (0.001 to 0.16)	0.04
*Covariates*				
Age, 1 yr older	-0.0003 (-0.001 to 0.001)	0.59	0.001 (-0.00007 to 0.002)	0.06
Female vs. Male	-0.19 (-0.22 to -0.16)	<0.001	-0.15 (-0.20 to -0.11)	<0.001
Race-Ethnicity				
Mexican American vs. White	-0.02 (-0.07 to 0.02)	0.35	-0.03 (-0.08 to 0.01)	0.21
Black vs. White	-0.02 (-0.07 to 0.01)	0.24	-0.05 (-0.10 to -0.004)	0.03
Other vs. White	-0.01 (-0.09 to 0.06)	0.71	-0.05 (-0.15 to 0.05)	0.35
BMI, 1 kg/m^2^ higher	-0.006 (-0.01 to -0.002)	0.006	-0.004 (-0.006 to -0.001)	0.003
1+ Comorbidities vs. None	0.003 (-0.04 to 0.04)	0.88	0.02 (-0.02 to 0.06)	0.33
Cotinine, 1 ng/mL higher	-0.0002 (-0.0003 to -0.00005)	0.01	-0.0001 (-0.0002 to 0.00007)	0.25
PIR, 1 unit higher	-0.001 (-0.01 to 0.01)	0.86	0.002 (-0.01 to 0.01)	0.74

^†^2 multivariable linear regression models were computed; 1 for insulin sensitive participants and another for insulin resistant individuals.

^‡^Expressed as a 30 unit/min change.

MVPA = Moderate-to-vigorous physical activity.

BMI = Body mass index.

PIR = Poverty-to-Income Ratio.

## Discussion

The major finding of this study was that accelerometer-determined MVPA was positively associated with bilirubin among insulin resistant adults, but not insulin sensitive adults. Among insulin resistant individuals, for every 30 minute increase in MVPA, there was a 0.08 mg/dL increase in bilirubin. As reported elsewhere [13], bilirubin changes of this magnitude (i.e., < 0.1 mg/dL) have been shown to associate with an approximate 3% decreased risk of peripheral vascular disease, 4% reduced risk of stroke, and a 5% reduced risk of cardiovascular disease. This study is novel, as, to our knowledge, no studies to date have examined the association between objectively-measured physical activity and bilirubin.

At the time of this writing, we are aware of only 3 other studies that have examined the independent effects of physical activity on bilirubin levels. In 2008, Devries et al. [12] reported no association between cycling training and bilirubin levels in lean or obese adults. In 2012, Swift et al. [13], did, however, demonstrate a positive association between 6 months of aerobic exercise training and bilirubin levels among insulin resistant adults, with no association found for insulin sensitive adults. Most recently, in 2013, Tanaka et al. [14] reported no association between self-reported physical activity and bilirubin levels.

Our findings are similar to those of Swift et al. [13] in that physical activity was associated with bilirubin for insulin resistant but not insulin sensitive adults. It is difficult to explain why physical activity was only associated with bilirubin among insulin resistant adults. A possible explanation, however, is that insulin resistant individuals had lower bilirubin levels when compared to insulin sensitive individuals, which may allow for greater change in bilirubin from physical activity.

Our findings do differ from those of Devries et al. [12] and Tanaka et al. [14] who reported null findings between physical activity and bilirubin. Although speculative, the null findings reported by Devries et al. [12] and Tanaka et al. [14] may be, in part, from the methodology employed. Tanaka et al. [14] utilized self-report physical activity methodology, which may have attenuated the association toward the null given the considerable measurement error associated with self-report physical activity [23]. The null findings by Devries et al. [12] may also have been a result of the mode of exercise (i.e., cycling). Increased heel-strike, weight bearing physical activity may facilitate increased hemooxygnase-1 activity (HO-1) [24], which is the enzyme responsible for the conversion of biliverin to bilirubin. In addition to increases in HO-1 activity, other potential mechanisms include weight-bearing physical activity-induced hemolysis (due to increased heel strike) [13,24]. Speculatively, cycling training may not be sufficient enough to increase the activity of the HO-1 system or induce hemolysis.

Limitations of the present study include the cross-sectional design, rendering causal inferences not possible. Additionally, and although total bilirubin are linked with cardiovascular risk [2,25,26], it was not possible to assess other components of bilirubin, such as free, conjugated or unconjugated bilirubin. Further, insulin sensitivity and insulin resistance was determined using the surrogate HOMA-IR method, as opposed to, for example, the hyperinsulinemic euglycemic clamp method. Despite these limitations, major strengths of this study include an investigation of this understudied topic, using an objective measure of physical activity, and employing a nationally representative sample of U.S. adults.

In summary, our analyses demonstrated a positive association between accelerometer-assessed MVPA and total bilirubin among adults with evidence of insulin resistance. Future experimental and prospective studies are needed, with further attention focused on the mechanisms that may help to explain the association between physical activity and bilirubin.

## Competing interests

All authors declare no conflicts of interest.

## Authors’ contributions

Both authors made substantive contributions to the conception of the study, interpretation of the data, were involved in drafting or revising the manuscript, have given final approval of the version to be published, and agree to be accountable for all aspects of the work.
